# Right Ventricular Dysfunction Predicts Outcome in Acute Heart Failure

**DOI:** 10.3389/fcvm.2022.911053

**Published:** 2022-05-18

**Authors:** Max Berrill, Eshan Ashcroft, David Fluck, Isaac John, Ian Beeton, Pankaj Sharma, Aigul Baltabaeva

**Affiliations:** ^1^Department of Cardiology, St. Peter’s Hospital, Surrey, United Kingdom; ^2^Department of Research and Development, St. Peter’s Hospital, Surrey, United Kingdom; ^3^Institute of Cardiovascular Research, Royal Holloway, University of London, Egham, United Kingdom; ^4^Department of Cardiology, Royal Brompton and Harefield Hospital, London, United Kingdom

**Keywords:** acute heart failure, RV failure, RV dysfunction, ejection fraction, strain, strain rate

## Abstract

**Aim:**

The severity of cardiac impairment in acute heart failure (AHF) predicts outcome, but challenges remain to identify prognostically important non-invasive parameters of cardiac function. Left ventricular ejection fraction (LVEF) is relevant, but only in those with reduced LV systolic function. We aimed to assess the standard and advanced parameters of left and right ventricular (RV) function from echocardiography in predicting long-term outcomes in AHF.

**Methods:**

A total of 418 consecutive AHF patients presenting over 12 months were prospectively recruited and underwent bedside echocardiography within 24 h of recruitment. We retrospectively assessed 8 RV and 5 LV echo parameters of the cardiac systolic function to predict 2-year mortality, using both guideline-directed and study-specific cutoffs, based on the maximum Youden indices *via* ROC analysis. For the RV, these were the tricuspid annular plane systolic excursion, RV fractional area change, tissue Doppler imaging (TDI) peak tricuspid annular systolic wave velocity, both peak- and end-systolic RV free wall global longitudinal strain (RV GLS) and strain rate (mean RV GLSR), RV ejection fraction (RVEF) derived from a 2D ellipsoid model and the ratio of the TAPSE to systolic pulmonary artery pressure (SPAP). For the LV, these were the LVEF, mitral regurgitant ΔP/Δt (MR dP/dt), the lateral mitral annular TDI peak systolic wave velocity, LV GLS, and the LV GLSR.

**Results:**

A total of 7/8 parameters of RV systolic function were predictive of 2-year outcome, with study cutoffs like international guidelines. A cutoff of < −1.8 s^–1^ mean RV GLSR was associated with worse outcome compared to > −1.8 s^–1^ [HR 2.13 95% CI 1.33–3.40 (*p* = 0.002)]. TAPSE:SPAP of > 0.027 cm/mmHg (vs. < 0.027 cm/mmHg) predicted worse outcome [HR 2.12 95% CI 1.53–2.92 (*p* < 0.001)]. A 3-way comparison of 2-year mortality by LVEF from the European Society of Cardiology (ESC) guideline criteria of LVEF > 50, 41–49, and < 40% was not prognostic [38.6% vs. 30.9 vs. 43.9% (*p* = 0.10)]. Of the 5 parameters of LV systolic function, only an MR dP/dt cutoff of < 570 mmHg was predictive of adverse outcome [HR 1.63 95% CI 1.01–2.62 (*p* = 0.047)].

**Conclusion:**

With cutoffs broadly like the ESC guidelines, we identified RV dysfunction to be associated with adverse prognosis, whereas LVEF could not identify patients at risk.

## Introduction

Acute heart failure (AHF) is a leading cause of hospitalization (1, 2) and carries a substantial risk of short- (3) and long-term mortality (4, 5). Echocardiography remains an essential tool in the evaluation of systolic function in all hospitalized AHF patients (6) and can provide crucial management-changing information with swift bedside hemodynamic assessment.

Left ventricular ejection fraction (LVEF) is routinely used as a surrogate for global left ventricular (LV) performance, but the evaluation of LV function remains challenging. Strain imaging has confirmed that a preserved EF does not guarantee “normal” systolic function (7, 8) and there is a highly complex relationship between EF and mortality (9–11). Poor outcomes are observed in those with significantly impaired EF (i.e., < 35–40%) (10, 12) but are also seen in the older (13), more comorbid (14) population with preserved ejection fraction (i.e., > 50%).

This complexity is reflected in the changes within international guidelines which have aimed to identify prognostic LVEF cutoff values, including the recent incorporation of a “mildly reduced ejection fraction” within European guidelines (6, 15) and “borderline/improved” within American guidelines (16). However, the evidence for all the current cutoffs is derived from trials investigating neurohormonal downregulation (17–19) or cardiac resynchronization therapy in *chronic* HF (20). The role of LVEF in AHF remains poorly investigated.

Historically the importance of right ventricular (RV) dysfunction has trailed the LV (21, 22) despite growing evidence that RV systolic dysfunction is an independent predictor of outcome across a range of LVEF (23–27). In fact, in 2006, the underrepresentation of the RV prompted the convocation of a working group by the National Heart, Lung, and Blood Institute to highlight and promote the contemporary understanding of its importance in heart failure (HF) (21).

We hypothesized that RV, rather than LV, systolic dysfunction may be more closely associated with long-term outcomes in AHF. To investigate this, we retrospectively used the study cohort of a prospective, observational study—the Mitral Regurgitation in Acute Heart Failure (MRAHF) study—to evaluate a battery of guideline-suggested and innovative assessments of systolic function of both RV and the LV.

## Materials and Methods

### Outline

The MRAHF study methods are previously published (28). In summary, this study was a prospective, observational cohort investigation over 12 months at a single center. A total of 418 consecutive individuals who presented with signs of heart failure and met objective criteria of AHF with raised point-of-care BNP level > 100 pg/ml were enrolled and all patients underwent comprehensive bedside echocardiography within 24 h of recruitment to confirm AHF. Alternative diagnoses were excluded with patients presenting with sepsis, pulmonary respiratory failure, and chronic heart failure excluded.

Participants were followed up for 24 months and assessed by all-cause mortality through the United Kingdom summary care record system and by the online software Evolve™ (Kainos, United Kingdom) for patient records, including death certificates, used at our hospital.

Trial oversight was by the Ashford and St Peter’s NHS Trust Research and Development team and was approved by the institutional review board and ethics committee. All patients gave written informed consent before enrollment in the study. All authors had access to data and this manuscript for review. The experimental design and decision for publication were by Dr. A. Baltabaeva.

### Echocardiography

Echocardiography was carried out with G.E. Vivid S70 (GE Healthcare, United States) and analyzed and stored using EchoPac v202.5 (GE Healthcare, United States). Exams were performed with a dedicated protocol (Supplementary Appendix 1). Off-line measurements were carried out by two echocardiographers with the British Society of Echocardiography (BSE) level II transthoracic echo (TTE) accreditation. Studies and measurements were cross-referenced by a consultant cardiologist with > 10 years of practice as an imaging expert, based at the Department of Echocardiography for a high-volume cardiothoracic surgical, transplant and tertiary referral center with the European Association of Cardiovascular Imaging (EACVI) accreditation.

The assessment of the left and right atrial and ventricular geometry, RV and LV systolic, and data on calculation of systolic pulmonary artery pressures were obtained using a standard TTE minimum dataset approach advocated by the BSE (29).

### Right Ventricular Systolic Parameters

RV systolic function assessment parameters included were the tricuspid annular plane systolic excursion (TAPSE); RV Fractional Area Change (RV FAC); RV Tissue Doppler Imaging (TDI) peak systolic wave velocity (RV S’); two-dimensional RV ellipsoid ejection fraction (RVEF) (30); RV Global Longitudinal Strain (RV GLS), and mean strain rate (RV GLSR) as well as the TAPSE to Systolic Pulmonary Artery Pressure Ratio (TAPSE:SPAP).

The RV GLS was assessed by taking the mean of the peak systolic and end-systolic strain from the basal, mid, and apical RV free wall by off-line analysis on the EchoPac workstation (v202.5) using LV dedicated strain analysis. RV systole was defined as the period between pulmonary valve opening and closure. Deformation of the interventricular septum was not included. RV GLSR was obtained by averaging values from the peak strain rate before pulmonary valve closure and from the basal, mid, and apical free wall segments.

LV systolic function assessment parameters included the LVEF; LV mitral regurgitation ΔP/Δt (LV MR dP/dt) when available; LV TDI lateral mitral annular peak systolic wave velocity (LV S’); LV Global Longitudinal Strain (LV GLS), and strain rate (LV GLSR).

### Statistical Analysis

Statistical analysis was carried out using MedCalc v.20.015 (MedCalc^®^ Software Ltd., Belgium). Receiver Operator Curve (ROC) analyses were carried out on the previously discussed RV and LV systolic function parameters. The optimum cutoff for the prediction of 24-month mortality was estimated by identifying the sensitivity and specificity associated with the maximum Youden Index.

A 24-month mortality analysis was carried out by constructing unstratified Kaplan–Meier survival curves using ROC and guideline cutoffs. Hazard ratios were estimated using an unadjusted Cox regression model, with statistical significance being assessed using the Log-rank test. For all statistical comparisons in this study, significance was defined as a 2-sided α-value of 0.05; 4 patients were lost to follow-up leaving 414/418 (99.0%) with available 2-year mortality data. Patients lost to follow-up were omitted from KM analysis.

Patients with poor acoustic windows and unmeasurable parameters were also omitted from the analysis. The numbers available for each RV parameter are as follows: TAPSE 411/414, RVFAC 409/414, SPAP 401/414, TAPSE/SPAP 398/414, 2D RVEF 412/414, RV end-systole GLS 346/414, RV peak GLS 346/414, RV average GLSR 346/414 and RV S’ 398/414. For the LV: LVEF 411/414, LV S’ 404/414, LV MR dP/dT 352/414, LV GLS 411/414 and LV GLSR 411/414.

If the unadjusted Cox regression model Log-rank tests from the ROC analysis failed to meet the 2-sided α-value of 0.05, further Cox regression models were constructed using ESC-guideline-specified thresholds, if not previously carried out (i.e., LVEF). Once again, statistical significance was assessed using the Log-rank test.

To determine whether the impact of LV and RV systolic assessment changes, and is independently associated with outcome, when analyzed as continuous variables instead of categorical variables with cutoffs, we constructed logistic regression models including 8 relevant cardiovascular demographic and clinical comorbidities previously included in the original results of the MRAHF study (28). These were age, gender, body mass index (BMI), previous history of cerebrovascular accident (CVA) or coronary artery disease, and a prior diagnosis of hypertension, diabetes mellitus, or chronic pulmonary obstructive disease (COPD).

These models included 2 continuous, global assessments of LV and RV systolic function, LVEF and RV FAC, or LVEF and RVEF. We included both a guideline-directed assessment (RVEF) and a more experimental assessment (2D RVEF) to cross-reference the importance of RV global systolic performance with any method of assessment. In these models, the dependent variable was the binary outcome of survival vs. mortality at 2 years. The Enter method was used where all variables were included in a single step. The significance level was defined at a threshold of 0.05 and the odds ratio was calculated with 95% confidence intervals.

## Results

**FIGURE 1:**
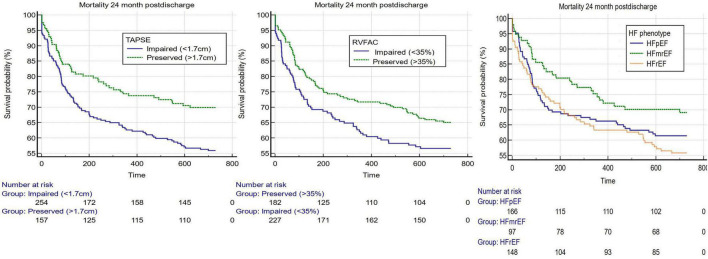
Kaplan–Meier estimates 3 guideline-suggested cutoffs of the most used systolic assessments of both the right and left ventricles. From left to right, TAPSE (cutoff 1.7 cm) (*p*-value = 0.0051), RV FAC (cutoff 35%) (*p*-value = 0.049), and LVEF (cutoffs for heart failure with preserved ejection fraction (LVEF > 50%), heart failure with mildly reduced ejection fraction (LVEF 41–49%) and heart failure with reduced ejection fraction (LVEF < 40%) (*p*-value = 0.109).

Using the ESC guideline-suggested cutoffs of RV and LV systolic function, Kaplan–Meier estimates were constructed and are displayed in Figure 1. The cutoff of 1.7 cm for TAPSE was associated with a worse 2-year prognosis for impaired vs. preserved longitudinal RV systolic function [HR 1.57 95% CI 1.15–2.16 (*p* = 0.005)]. The cutoff of 35% for RV FAC was also associated with a worse 2-year prognosis for impaired vs. preserved FAC [HR 1.38 95% CI 1.00–1.89 (*p* = 0.049)]. Using an ESC-suggested 3-phenotype model of HF, LVEF was assessed in a 3-way comparison of > 50, 41–49, and < 50%, but these cutoffs were not significantly associated with outcome (Figure 1 and Supplementary Table 1).

Assessments of RV and LV systolic dysfunction *via* ROC analyses (Supplementary Table 2 and Supplementary Figures 1, 2) identified the maximum Youden index-associated criteria which were compared with guideline-suggested cutoffs where available (Table 1). There were broad similarities between the cutoffs derived from the MRAHF data ROC analyses and the guideline-recommended cutoffs, apart from the LV GLS. The greatest differences between the MRAHF and guideline-discussed cutoffs were for LV GLS (−6.32 vs. c.−20%), which might be a reason the latter did not make into an official cutoff in chamber quantification release.

**TABLE 1:** Assessments of right ventricular (RV) and left ventricular (LV) systolic dysfunction.

Systolic assessment	Younden index cutoff	Guideline binary cutoff
**Right ventricle**		
*TAPSE (cm) (n* = *411)*	1.6	1.7
*RV FAC (%) (n* = *409)*	38.2	35
*RV S’ (m/s) (n* = *398)*	0.09	0.095
*2D RVEF (%) (n* = *412)*	46.9	n/a
*RV peak GLS (%) (n* = *346)*	−18.6	−20
*RV end-systole GLS (%) (n* = *346)*	−18	n/a
*Mean RV GLSR (s^–1^) (n* = *346)*	−1.8	n/a
*TAPSE:SPAP (cm/mmHg) [n* = *398]*	0.0268	n/a
***Left Ventricle***		
*LVEF (%) (n* = *411)*	48	50*
*LV S’ (m/s) (n* = *404)*	0.06	n/a
*LV GLS (%) (n* = *411)*	−6.32	n/a
*LV GLS rate (s^–1^) (n* = *411)*	−0.86	n/a
*LV MR dp/dt (mmHg/s) (n* = *352)*	570	n/a

*ESC guidelines identify 50% as the cutoff for preserved EF but they do not suggest a binary cutoff for LVEF and instead delineate into 3 phenotypes - heart failure-preserved ejection fraction, heart failure with mildly reduced ejection fraction, heart failure with reduced ejection fraction. TAPSE, tricuspid annular plane systolic excursion; RV FAC, RV Fractional Area Change; RV S’, RV Tissue Doppler Imaging (TDI) tricuspid annular peak systolic wave velocity; 2D RVEF, two-dimensional RV ellipsoid ejection fraction; RV inferior wall GLS, RV Inferior Wall Global Longitudinal Strain; TAPSE:SPAP, TAPSE to Systolic Pulmonary Artery Pressure Ratio; LVEF, left ventricular ejection fraction; LV S’, LV TDI lateral mitral annular peak systolic wave velocity; LVGLS, LV global longitudinal strain; LV MR dp/dt, LV GLS rate (LV GLSR) and LV mitral regurgitation Δp/Δt.

**FIGURE 2:**
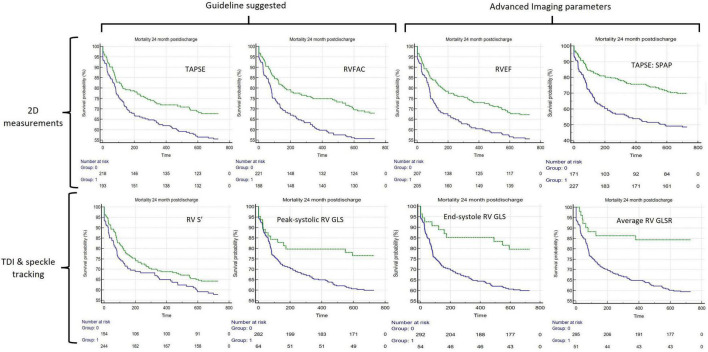
Unadjusted Kaplan–Meier curves comparing 8 different assessments of RV systolic function, in all. The green lines represent the unimpaired systolic function and blue lines represent impaired systolic function. The echo parameter assessment of systolic function is displayed in the top-right corner of each (clockwise from top-left): TAPSE ≤ 1.6 cm, RV FAC ≥ 38.2%, 2D ellipsoid RVEF < 46.9%, TAPSE:SPAP < 0.0268 (cm/mmHg). RV S’ ≤ 0.09 m/s, Peak systolic RV GLS > −18.6%, End-systole RV GLS > −18% and RV mean GLSR > −1.8 s^–^ TAPSE, Tricuspid Annular Plane Systolic Excursion; RV FAC, RV Fractional Area Change; RV S’ RV Tissue Doppler Imaging peak systolic velocity; RVEF, two-dimensional RV ellipsoid ejection fraction; RV GLS, RV Free Wall Global Longitudinal Strain; mean RV GLSR, mean RV free wall global longitudinal strain rate; TAPSE:SPAP, TAPSE to Systolic Pulmonary Artery Pressure Ratio.

Of the 8 RV parameters assessed, 7 were significantly associated with worse outcomes (Table 2). The RV GLSR > −1.8 s^–1^ vs. < −1.8 s^–1^ exhibited the highest hazard ratio of 2.13 [95% CI 1.33–3.40 (*p* = 0.002)]. The only RV parameter which was not significantly associated with a worse outcome was the RV S’ velocity ≤ 0.09 m/s vs. > 0.09 m/s (*p* = 0.170). The unadjusted Kaplan–Meier curves of these cutoffs are displayed in Figure 2. An ESC guideline abnormality threshold of 0.095 m/s Lang et al. (31) was then assessed but was not significantly associated with the outcome (*p* = 0.169) (Supplementary Table 3).

**TABLE 2:** Assessments of right ventricular (RV) and left ventricular (LV) systolic dysfunction with binary cutoffs as determined by the criteria associated with the maximum Youden index.

Systolic assessment	Binary cutoff	Hazard ratio (95% CI)	*p*-value
**Right ventricle**			
*TAPSE (cm)*	≤1.6	1.50 (1.10–2.05)	**0.011**
*RV FAC (%)*	≤38.2	1.54 (1.13–2.11)	**0.007**
*RV TDI S wave velocity (m/s)*	≤0.09	1.26 (0.91–1.75)	0.170
*RV peak inferior free wall GLS*	>-18.6	1.67 (1.08–2.59)	**0.021**
*RV end-systole inferior free wall GLS (%)*	>-18	1.87 (1.18–2.95)	**0.008**
*RV mean GLS rate (s^–1^)*	>-1.8	2.13 (1.33–3.40)	**0.002**
*2D RVEF (%)*	≤46.9	1.50 (1.10–2.06)	**0.010**
*TAPSE:SPAP*	>0.0268	2.12 (1.53–2.92)	**<0.001**
***Left ventricle***			
*LV ejection fraction (%)*	>48	1.08 (0.78–1.50)	0.641
*LV TDI S wave velocity (m/s)*	≤0.06	1.22 (0.88–1.79)	0.231
*LV GLS (%)*	>-6.32	1.25 (0.88–1.79)	0.210
*LV GLS rate (s^–1^)*	≤-0.86	1.28 (0.89–1.83)	0.186
*LV MR dp/dt (mmHg)*	≤570	1.63 (1.01–2.62)	**0.047**

*Hazard ratios indicate the hazard ratio associated with all-cause mortality at 2 years constructed from unadjusted Cox regression analysis, with p-values determined from the Log-rank test. Tricuspid Annular Plane Systolic Excursion (TAPSE); RV Fractional Area Change (RV FAC); RV Tissue Doppler Imaging systolic velocities (RV TDI S wave velocity), two-dimensional RV ellipsoid ejection fraction (2D RVEF); RV Inferior Wall Global Longitudinal Strain (RV inferior wall GLS); TAPSE to Systolic Pulmonary Artery Pressure Ratio (TAPSE:SPAP); LV mitral regurgitation Δp/Δt (LV MR dp/dt). p-values in bold are statistically significant according to our threshold of 0.05.*

**FIGURE 3:**
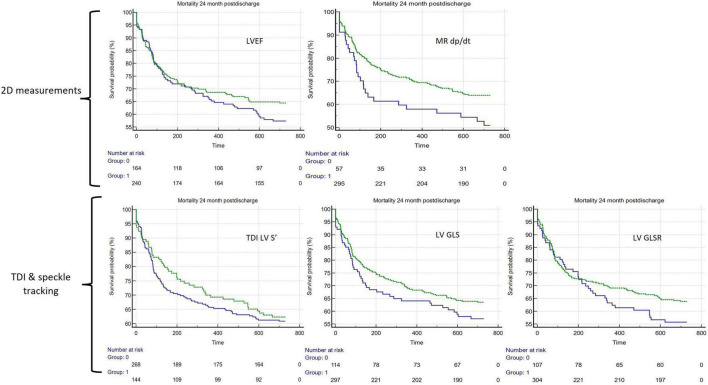
Unadjusted Kaplan–Meier curves comparing 5 different assessments of LV systolic function. In all panels, green lines represent preserved systolic function and blue lines represent impaired function (clockwise from top-left): LVEF > 48%, LV MR dp/dt < 570 mmHg/s, LV TDI S’ ≤ 0.06 m/s, LV GLS > −6.32%, LV GLSR > −0.86 s^–1^. LVEF, LV ejection fraction; LV MR dp/dt, LV mitral regurgitation Δp/Δt; LV S’, LV Tissue Doppler Imaging (TDI) lateral mitral annular peak systolic velocity; LV GLS, LV Global Longitudinal Strain; LV GLSR, LV global longitudinal strain rate.

Of the binary cutoffs of the 5 LV parameters only the MR dP/dt < 570 mmHg vs. > 570 mmHg was significantly associated with a worse outcome, hazard ratio 1.62 [95% CI 1.01–2.62 (*p* = 0.047)] (Table 2). The unadjusted Kaplan–Meier estimates of these cutoffs are displayed in Figure 3.

RV FAC and LVEF were included in a logistic regression model as continuous variables alongside 8 cardiovascular comorbidities previously included in the MRAHF 2-year outcome study (28) (Supplementary Table 4). RV FAC was an independent predictor of outcome (coefficient −0.02, odds ratio 0.98 [95% CI 0.96–1.00 (*p* = 0.028)] and LVEF was not (*p* = 0.794). Age, CKD, and COPD were also independent predictors of outcome. The model area under the ROC curve (AUC) was 0.744 (95% CI 0.699–0.786). To investigate if this effect was limited only to FAC, we also included another global assessment of RV systolic function with the ellipsoid assessment of RV ejection fraction, which was also an independent predictor of outcome OR 0.98 [95% CI 0.96–1.00 (*p* = 0.01)] (Supplementary Table 5).

## Discussion

This study characterized the prognostic impact of a battery of RV and LV systolic non-invasive assessments by echocardiography. The study-specific cutoffs for 13 parameters of systolic RV and LV function were broadly like the ESC guidelines, except for LV GLS. Overall, this study suggests that poor RV function determines outcome in AHF.

Most RV systolic assessments were associated with adverse outcome at a 2-year follow-up and this study confirmed the prognostic strength of standard parameters of RV function such as TAPSE and RVFAC which have well-established evidence in risk stratification for chronic heart failure patients (32–35). This study extends the more limited evidence for their role in prognosis in the setting of AHF (36) and adds weight to the application of current guideline cutoffs in this context.

RV longitudinal deformation obtained from the speckle-tracking technique has already been proven to be of prognostic importance despite load dependency (37) across a range of pathologies (38). RV strain proved to be a feasible and reproducible echocardiographic technique available in 83.5% of our cohort, all of whom were acutely unwell and breathless. It provided important additional information on RV mechanics which may be because RV longitudinal shortening is more important for systolic function than circumferential shortening (39).

In this analysis, we used RV free wall strain rather than RV global strain to exclude the septum which is largely influenced by LV myocardial function (31). Meanwhile, the strain rate, a non-invasive measure of myocardial contractility (40), was complimentary to deformation parameters and was able to discriminate those at risk of worse outcomes despite known problems with noise (41). To our knowledge, this is the first study to assess the role of RV GLSR in AHF. Given that our study data correspond well to international guidelines for RV systolic assessment, the study-specific cutoff of −1.8 s^–1^ may be of relevance to further research on the role of echo assessment of RV strain rate analysis.

To test the importance of RV function, we applied several other innovative assessments of RV systolic function which are not part of international guidelines. We applied a two-dimensional ellipsoid model to estimate RVEF which has previously been confirmed to be non-inferior to TAPSE and RV FAC in this cohort of patients (30). In our logistic regression model, it was independently associated with mortality.

We also included the TAPSE/SPAP ratio, a relatively novel estimate of the right ventricular-vascular coupling to adjust to afterload caused by the left heart disease. It has been suggested to predict the outcome in heart failure with preserved ejection fraction (HFpEF) (22) and pulmonary hypertension (42). In unadjusted analysis, our study data confirmed it as an additional value as a non-invasive prognostic parameter within the broad “all-comers” AHF setting of this study.

Given the good correlation of our study dataset to international guideline cutoffs, the cutoffs for more novel assessments such as 2D RVEF and TAPSE/SPAP ratios provided here may be of relevance for further research. Subject to confirmation by future studies in AHF, we think that 2D echo-derived RVEF and the SPAP/TAPSE ratio have the potential to play a significant role in clinical practice given both the ease of echo data acquisition and their potential prognostic significance.

RV S’ was the only right-sided parameter that failed to reliably predict the outcome. This may be the result of angle-dependency which becomes an issue with RV remodeling and image acquisition in an acute setting where time limitations for scanning the unwell patient do not allow correct positioning of echo windows.

Unlike RV function parameters, LVEF and most of the LV systolic function parameters were not associated with the outcome when used either as a binary cutoff, or in a guideline-directed 3-way comparisons of HFpEF, heart failure with mildly reduced ejection fraction (HFmrEF), and heart failure with reduced ejection fraction (HFrEF).

The evaluation of ESC-suggested cutoffs for HFpEF, HFmrEF, and HFrEF echoed the results of retrospective analysis of the ASCEND-HF study (11) with a pattern of similarly poor, unadjusted, outcomes between HFrEF and HFpEF, with a slightly better prognosis of HFmrEF. Because of this complex relationship, LVEF remains a poor tool to predict outcomes in the setting of AHF, where there is a heterogeneous patient population who may present with advanced diastolic dysfunction, pulmonary hypertension, RV failure, or a combination of these, as examples. We feel the overly simplistic approach to relying on LVEF to assess global cardiac performance is outdated.

In the most recent guidelines of chamber quantification from EACVI/ESC, a value of −20% is identified to be suggestive of “healthy” myocardial strain (31) but no clear cutoff exists to identify poor LV longitudinal deformation, in part due to the heterogeneity of vendor and software measurements. In this study, a cutoff was substantially lower than what the guidelines suggest is “healthy” (−6%) and could not delineate those at risk of poor outcome. The role of GLS in AHF remains an important avenue for further investigation.

The only assessment of LV systolic function which displayed a discriminatory capacity for outcome was the MR dP/dt. This is a relatively load-independent reflection of global left ventricular contractility (43) which corresponds to the instantaneous pressure difference between the left atrium and LV (44). The cutoff suggested from the ROC analysis (570 mmHg) is broadly in keeping with the cutoff of 600 mmHg/s (45), indicating “severe LV dysfunction” because of advanced myocardial disease.

### Limitations

There are several limitations of this study. It was conducted at a single center which limits the generalizability of its findings and further confirmatory studies are warranted. We have not further investigated LVEF as a continuous variable in the sub-set of individuals with significantly impaired EF (i.e., < 40%), where there is evidence for worsening outcome as LVEF deteriorates further (12). However, the worse outcome associated with severely low MR dP/dt (< 570 mmHg) is indicative of this.

Echo assessment of RV strain was adapted from the LV strain analysis software with timing used from pulmonary valve opening and closure, ideally, an LV-specific software should be used. We have also not carried out a multivariate analysis of every RV parameter which was shown to be predictive of the outcome of an unadjusted assessment. However, we selected two global assessments of RV systolic function (RVFAC and RVEF) and performed logistic regression analysis with cofounders selected from our previous MRAHF study results. We selected RVFAC as a guideline-directed and RVEF as a more experimental parameter.

### Summary

In patients presenting with AHF, RV decompensation, confirmed by both load-dependent and independent parameters, is the main determinant of a long-term outcome. We have tested the novel parameters of RV function which proved to be feasible and significant as additional tools for standard RV assessment. This study suggests that a combination of left heart-induced afterload and depleted RV myocardial reserve plays an important role. This highlights the need to move past an overly simplistic reliance on the LV ejection fraction when assessing cardiac performance in AHF. More attention should be paid to LV dP/dt when the MR jet is available for accurate assessment.

## Data Availability Statement

The original contributions presented in the study are included in the article/Supplementary Material, further inquiries can be directed to the corresponding author/s.

## Ethics Statement

The studies involving human participants were reviewed and approved by Ashford and St. Peter’s NHS Trust Institutional Review Board and Ethics Committee. The patients/participants provided their written informed consent to participate in this study.

## Author Contributions

AB developed the experimental design with inputs from IB, DF, IJ, and PS. MB and AB wrote the manuscript with editing available to all authors. MB carried out the statistical analysis. AB and MB made the final decision for submission. All authors had access to the manuscript and data before publication.

## Conflict of Interest

The authors declare that the research was conducted in the absence of any commercial or financial relationships that could be construed as a potential conflict of interest.

## Publisher’s Note

All claims expressed in this article are solely those of the authors and do not necessarily represent those of their affiliated organizations, or those of the publisher, the editors and the reviewers. Any product that may be evaluated in this article, or claim that may be made by its manufacturer, is not guaranteed or endorsed by the publisher.
