# Supplementary material for: Right Ventricular Dysfunction Predicts Outcome in Acute Heart Failure
Source: Front Cardiovasc Med. 2022 May 18;9:911053. doi: 10.3389/fcvm.2022.911053 (PMC9157539; doi:10.3389/fcvm.2022.911053)
Supplement: Supplementary file 1 [file Data_Sheet_1.docx]

| **Systolic Assessment** | |  |  |  |
| --- | --- | --- | --- | --- |
|  |  | **HFpEF (n=166)** | **HFmrEF (n=97)** | **HFrEF (n=148)** |
| **Deaths - n (%)** | | 64 (38.55) | 30 (30.93) | 65 (43.92) |
| **Data censored - n (%)** | | 102 (61.45) | 67 (69.07) | 83 (56.08) |
|  |  |  |  |  |
| **Hazard ratio** | |  |  |  |
|  | HFmrEF vs. HFpEF (95% CI) |  | 0.74 (0.50-1.09) | |
|  | HFrEF vs. HFpEF (95% CI) |  | 1.17 (0.81-1.67) | |
|  | Hfmref vs. HFrEF (95% CI) |  | 0.63 (0.42-0.95) | |
|  |  |  |  |  |
|  | p-value (Logrank) |  | 0.109 |  |

*Supplementary Table 1: 3 way comparison of ESC guideline thresholds for LVEF using unadjusted Cox regression analysis. 414/418 had available 2-year mortality data and 411/414 had LVEF data available.*


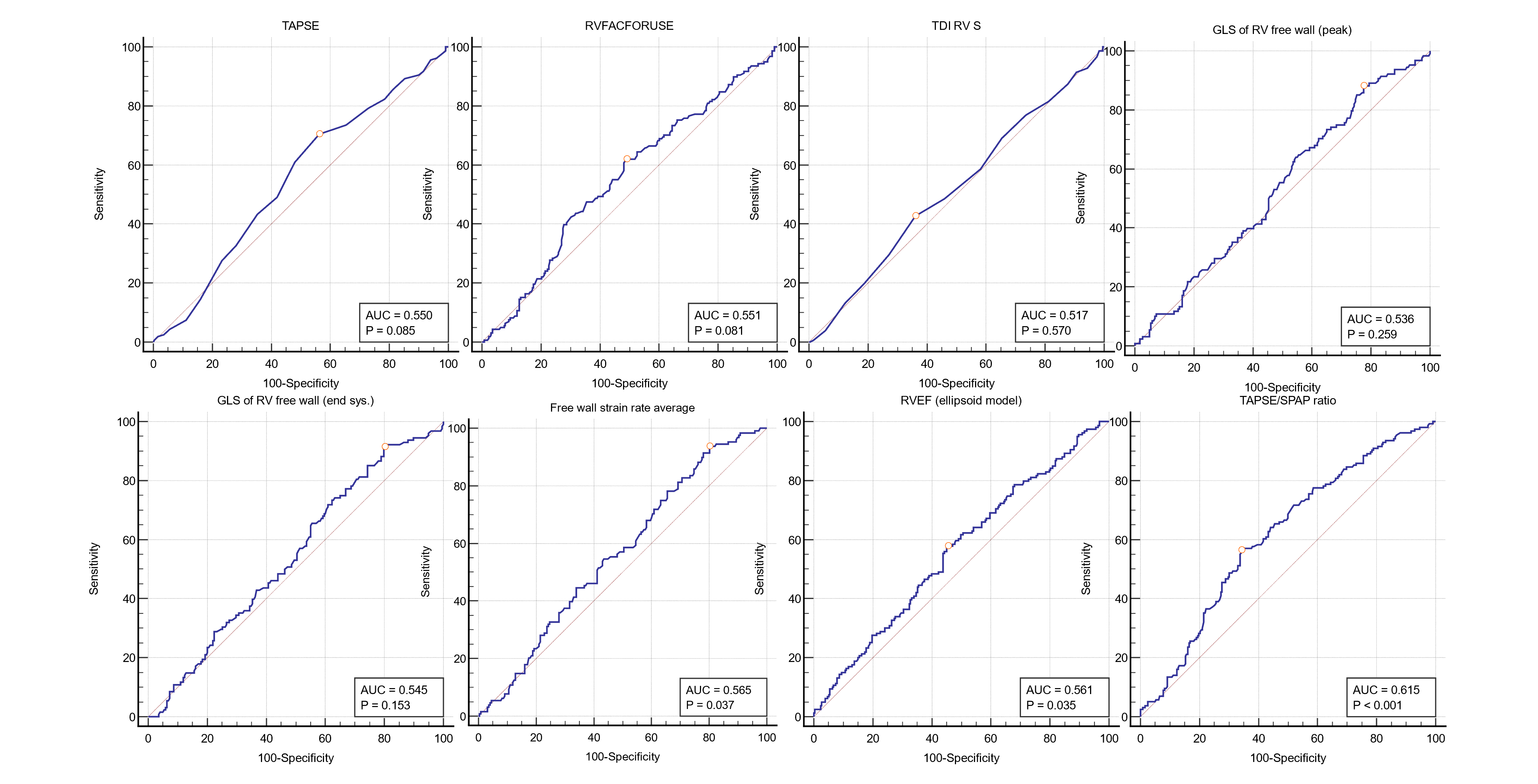


Supplementary Figure 1; receiver operator characteristic curves for RV systolic assessments.


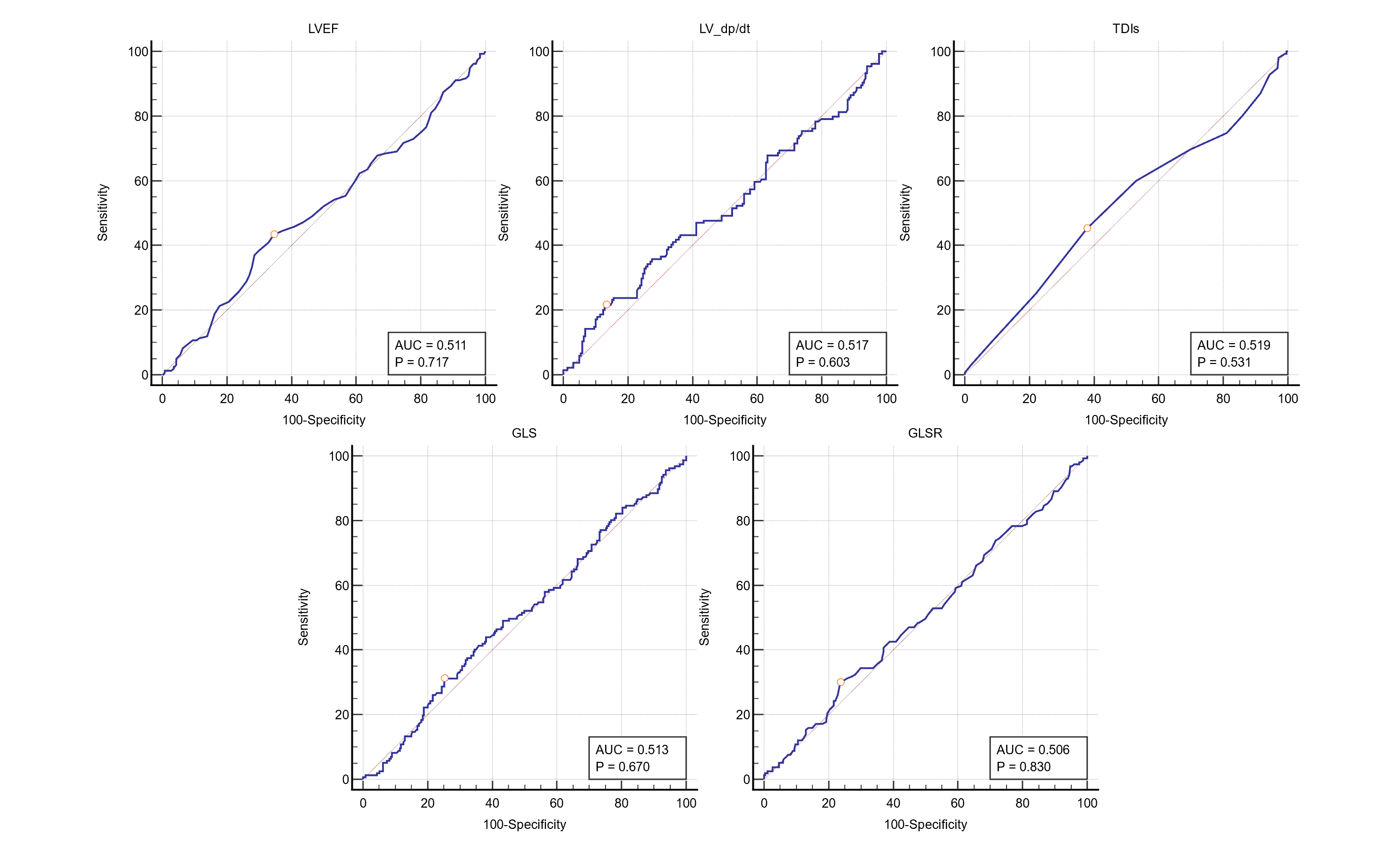


Supplementary Figure 2; receiver-operator characteristic curves for LV systolic assessments

| **Systolic Assessment** |  |  |  |  |
| --- | --- | --- | --- | --- |
|  | **Younden index J** | **Associated Criterion** | **Sensitivity (%)** | **Specificity (%)** |
| ***Right Ventricle*** |  |  |  |  |
|  |  |  |  |  |
| TAPSE (cm) | 0.141 | ≤1.60 | 70.4 | 43.7 |
| RV FAC (%) | 0.130 | ≤38.2 | 62.0 | 51.0 |
| RV TDI S wave velocity (m/s) | 0.067 | ≤0.09 | 42.8 | 63.8 |
| 2D RVEF (%) | 0.124 | ≤46.9 | 57.9 | 54.6 |
| RV GLS [peak] (%) | 0.108 | >-18.6 | 88.3 | 22.5 |
| RV GLS [end-systole] (%) | 0.111 | >-18.0 | 91.4 | 19.7 |
| RV strain rate average (cm/s) | 0.135 | >-1.80 | 93.8 | 19.7 |
| TAPSE:SPAP (cm/mmHg) | 0.221 | ≤0.027 | 56.4 | 65.7 |
|  |  |  |  |  |
| ***Left Ventricle*** |  |  |  |  |
|  |  |  |  |  |
| LV EF (%) | 0.089 | >48 | 43.4 | 65.5 |
| LV TDI S wave velocity (m/s) | 0.074 | ≤0.06 | 45.2 | 62.3 |
| LV GLS (%) | 0.060 | >-6.32 | 31.2 | 74.8 |
| LV GLS rate (cm/s) | 0.063 | ≤-0.86 | 29.9 | 76.4 |
| LV MR dp/dt (mmHg) | 0.083 | ≤570 | 21.6 | 86.7 |

*Supplementary Table 2: Receiver-operator characteristic analysis associated criteria for the maximum Younden indices with the associated sensitivities and specificities*

|  | Cut-off | Hazard ratio (95% CI) | p-value |
| --- | --- | --- | --- |
| ESC RV TDI S | 0.095m/s | 1.26 (0.91-1.75) | 0.169 |

Supplementary Table 3: Associated hazard ratio generated from unadjusted cox regression analysis for the European Society of Cardiography guideline suggested cut off for abnormal right ventricular tissue doppler imaging S wave velocity (<0.095m/s)

| **Variable** | **Coefficient** | **S.E.** | **Wald** | **p-value** | **OR** | **95% CI** |
| --- | --- | --- | --- | --- | --- | --- |
| Age | 0.073 | 0.013 | 32.6 | <0.001 | 1.075 | 1.049 to 1.103 |
| Male gender | -0.030 | 0.232 | 0.016 | 0.898 | 0.971 | 0.616 to 1.529 |
| Previous stroke/TIA | -0.526 | 0.325 | 2.63 | 0.105 | 0.591 | 0.313 to 1.116 |
| COPD | 0.974 | 0.313 | 9.70 | 0.002 | 2.649 | 1.435 to 4.892 |
| Coronary Artery Disease | 0.084 | 0.236 | 0.1265 | 0.722 | 1.087 | 0.685 to 1.725 |
| CKD | 0.701 | 0.230 | 9.2685 | 0.002 | 2.017 | 1.2838to 3.168 |
| Diabetes Mellitus | 0.188 | 0.250 | 0.5613 | 0.454 | 1.206 | 0.739 to 1.970 |
| Hypertension | -0.147 | 0.231 | 0.4053 | 0.524 | 0.863 | 0.549 to 1.357 |
| RVFAC | -0.021 | 0.009 | 4.8295 | 0.028 | 0.980 | 0.962 to 0.998 |
| LVEF | -0.002 | 0.008 | 0.06816 | 0.794 | 0.998 | 0.982 to 1.014 |
| Constant | -5.880 | 1.085 | 29.4002 | <0.001 | n/a | n/a |

Table 4: Logistic regression model to predict 2 year mortality. Transient ischaemic attack (TIA); chronic obstructive pulmonary disease (COPD), chronic kidney disease (CKD); right ventricular fractional area change (RVFAC); left ventricular ejection fraction (LVEF); standard error (S.E.); odds ratio (OR); confidence interval (CI)

| **Variable** | **Coefficient** | **S.E.** | **Wald** | **P** | **Odds ratio** | **95% Confidence Interval** |
| --- | --- | --- | --- | --- | --- | --- |
| Age | 0.067 | 0.012 | 29.635 | <0.0001 | 1.07 | 1.0439 to 1.0955 |
| Male gender | 0.008 | 0.230 | 0.001 | 0.97 | 1.01 | 0.6421 to 1.5810 |
| Previous stroke/TIA | -0.142 | 0.229 | 0.386 | 0.53 | 0.87 | 0.5538 to 1.3585 |
| COPD | -0.353 | 0.313 | 1.275 | 0.26 | 0.70 | 0.3808 to 1.2966 |
| Coronary Artery Disease | 1.005 | 0.312 | 10.390 | 0.00 | 2.73 | 1.4830 to 5.0365 |
| CKD | 0.108 | 0.234 | 0.214 | 0.64 | 1.11 | 0.7043 to 1.7635 |
| Diabetes Mellitus | 0.657 | 0.229 | 8.250 | 0.00 | 1.93 | 1.2321 to 3.0205 |
| Hypertension | 0.194 | 0.248 | 0.611 | 0.43 | 1.21 | 0.7466 to 1.9743 |
| LVEF | -0.001 | 0.008 | 0.011 | 0.92 | 1.00 | 0.9839 to 1.0147 |
| RVEF (ellipsoid model) | -0.021 | 0.008 | 6.222 | 0.01 | 0.98 | 0.9630 to 0.9955 |
| Constant | -5.282 | 1.069 | 24.417 | <0.0001 |  |  |

Supplementary Table 5: Logistic regression model of 8 cardiovascular comorbidities previously used in the Mitral Regurgitation in Acute Heart Failure study alongside left ventricular ejection fraction and a two dimensional ellipsoid model of the right ventricular ejection fraction. The outcome of interest was 2-year all-cause mortality.

**Supplementary Appendix 1**

MRAHF study protocol

| Patients admitted to ITU, HDU, Acute Medical Unit (AMU), coronary care unit (CCU), respiratory ward and care for elderly ward with pulmonary oedema, shortness of breath, clinical features of left and right heart congestion and/or palpitations.  Shortness of Breath (SOB), peripheral oedema, arrhythmias (AF, SVT, VT, Frequent VEs)  ꜜ ꜜ  No Yes  ꜜ ꜜ  No Further Action Clinical Examination and Phonocardiography  Systolic Murmur,  ꜜ ꜜ  Yes No  ꜜ ꜜ  i-STAT BNP i-STAT BNP  ꜜ ꜜ ꜜ ꜜ  Normal Elevated Elevated Normal  $\downarrow$ $\downarrow$ $\downarrow$ $\downarrow$  No Further TTE TTE No Further  Action $\downarrow$ $\downarrow$ Action  Grading of Mitral Regurgitation on TTE  $\downarrow$ $\downarrow$ $\downarrow$  0-2 3 4  $\downarrow$ $\downarrow$ $\downarrow$  No Further Action Retrospective Analysis of Notes |
| --- |
